# N-Octanoyl Dopamine Treatment of Endothelial Cells Induces the Unfolded Protein Response and Results in Hypometabolism and Tolerance to Hypothermia

**DOI:** 10.1371/journal.pone.0099298

**Published:** 2014-06-13

**Authors:** Eleni Stamellou, Johann Fontana, Johannes Wedel, Emmanouil Ntasis, Carsten Sticht, Anja Becker, Prama Pallavi, Kerstin Wolf, Bernhard K. Krämer, Mathias Hafner, Willem J. van Son, Benito A. Yard

**Affiliations:** 1 Vth. Medical Department, Medical Faculty Mannheim, Ruprecht Karls University Heidelberg, Mannheim, Germany; 2 Department of Neurosurgery, Ruhr-University Bochum, Bochum, Germany; 3 Medical Research Centre (ZMF), Medical Faculty Mannheim University of Heidelberg, Mannheim, Germany; 4 Institute for Molecular and Cellular Biology, Mannheim University of Applied Sciences, Mannheim, Germany; 5 Department of Nephrology, Academic Medical Centre, Groningen, the Netherlands; Medical University of South Carolina, United States of America

## Abstract

**Aim:**

N-acyl dopamines (NADD) are gaining attention in the field of inflammatory and neurological disorders. Due to their hydrophobicity, NADD may have access to the endoplasmic reticulum (ER). We therefore investigated if NADD induce the unfolded protein response (UPR) and if this in turn influences cell behaviour.

**Methods:**

Genome wide gene expression profiling, confirmatory qPCR and reporter assays were employed on human umbilical vein endothelial cells (HUVEC) to validate induction of UPR target genes and UPR sensor activation by N-octanoyl dopamine (NOD). Intracellular ATP, apoptosis and induction of thermotolerance were used as functional parameters to assess adaptation of HUVEC.

**Results:**

NOD, but not dopamine dose dependently induces the UPR. This was also found for other synthetic NADD. Induction of the UPR was dependent on the redox activity of NADD and was not caused by selective activation of a particular UPR sensor. UPR induction did not result in cell apoptosis, yet NOD strongly impaired cell proliferation by attenuation of cells in the S-G2/M phase. Long-term treatment of HUVEC with low NOD concentration showed decreased intracellular ATP concentration paralleled with activation of AMPK. These cells were significantly more resistant to cold inflicted injury.

**Conclusions:**

We provide for the first time evidence that NADD induce the UPR in vitro. It remains to be assessed if UPR induction is causally associated with hypometabolism and thermotolerance. Further pharmacokinetic studies are warranted to address if the NADD concentrations used in vitro can be obtained in vivo and if this in turn shows therapeutic efficacy.

## Introduction

The endoplasmic reticulum (ER) can be considered as the gatekeeper for protein synthesis, assuring appropriate protein folding and maturation of secreted and transmembrane proteins. These functions are highly regulated and require checkpoint for allowing that only correctly folded proteins can leave the ER. Whenever the folding capacity of the ER is too low to meet the cellular demand for newly synthesized proteins, unfolded or misfolded proteins start to accumulate in the ER, which in turn, triggers a set of responses known as the unfolded protein response (UPR) [1]–[4]. The function of the UPR can be divided into two major objectives, i.e. restoration of ER homeostasis and secondly induction of apoptosis if the first objective fails. The UPR accomplishes its function by increasing the expression of chaperone proteins to assist in protein folding, transient inhibition of mRNA translation to decrease protein influx into the ER, increasing ER associated degradation to remove misfolded proteins and induction of apoptotic genes to eliminate cells that are beyond repair.

The UPR consist of three different branches, each of which containing a specific transmembrane ER sensor protein that, upon activation, sets a series of responses in motion resulting in the transcription of typical UPR target genes [3], [4]. These so called UPR sensor proteins are the inositol requiring kinase 1 (IRE1), double-stranded RNA-activated protein kinase (PKR)-like ER kinase (PERK) and activating transcription factor 6 (ATF6) [1]–[3], all of which are associated with the ER chaperone glucose-regulated protein BiP. If misfolded proteins accumulate, BiP dissociates from the ER sensors, leading to their activation [1]–[3], [5]. UPR sensor activation is characterized by cleavage of X-box- binding protein 1 (Xbp1) mRNA (IRE1), phosphorylation of the translation initiation factor 2 α subunit (eIF2α) (PERK) and processing of ATF6 in the Golgi apparatus [6]–[12] The UPR has been implicated in a variety of diseases including cancer, metabolic, neurodegenerative and inflammatory diseases [13]. In addition it might be an integral part of the protective strategies used by hibernating mammals for long term survival in a state of cold torpor [14]. Signalling components of the UPR are emerging as potential targets for intervention and treatment of human disease [15].

Long chain polyunsaturated fatty acids are able to form conjugates with amines, e.g. ethanolamine, serotonin or dopamine. Since these conjugates can influence a variety of biological systems, they are gaining increased attention as promising new leads in the field of inflammatory and neurological disorders and for other pharmacological applications [16]. Endogenous N-acyl dopamines (NADD), e.g. N-arachidonoyl-, N-oleoyl-, N-palmitoyl- and N-stearoyl-dopamine are present in brain tissue and are known to modulate the endocannaboid system. It has also been reported that NADD are able to activate transient receptor potential (TRP) vanilloid subfamily member 1 (TRPV1) [17], [18]. We recently described N-octanoyl-dopamine (NOD), a short synthetic NADD, as a potent protective compound in acute kidney injury [19]. In addition it prevents hypothermic preservation injury [20], [21], strongly inhibits platelet function [22] and impairs NFκB activation [23]. Yet, it should be underscored that its increased hydrophobicity, as compared to dopamine, may allow NOD to cross intracellular membranes much easier where it may severely change the redox milieu within subcellular compartments [20]. Changes in redox milieu are either caused by donation of reduction equivalents as a consequence of quinone formation or by the iron chelating properties of the catechol structure [24]. In keeping with the importance of redox homeostasis in the ER for oxidative protein folding [25], [26], the present study was conducted to assess if synthetic N-acyl dopamine derivatives are able to activate the UPR. In addition we sought to identify the structural entities within NOD that might be important for UPR activation. To this end we made use of synthetic NADD that were either changed at the aromatic ring or in the aliphatic chain. We also sought to address if activation of the UPR by NOD compromises cell viability or if it represents a protective response, allowing cells to adapt to more aggravating conditions such as hypothermic preservation.

## Materials and Methods

### Reagents

Reagents were obtained from the sources as indicated: endothelial cell culture medium (Provitro, Berlin, Germany), PBS, trypsin solution, ethanol (GIBCO, Invitrogen, NY, USA), FBS Gold (PAA laboratories GmbH, Pasching, Austria), Actinomycin D, β-mercaptoethanol, tunicamycin, ethidium bromide, EDTA solution, DMSO, Tween 20, phospahatase inhibitor cocktail 2, Igepal 10%, collagenase, HEPES, Triton X-100, DTT, sodium deoxycholate, Tris-base, ammonium persulphate, SDS, TEMED, glycine, MTT, hexadimethrine bromide, acrylamid 40%, gelatine (Sigma-Aldrich, Taufkirchen, Germany), bovine serum albumin (SERVA, Heidelberg, Germany), protease inhibitor cocktail, first strand cDNA synthesis Kit (Roche Diagnostic, Mannheim, Germany), Dual-Glo Luciferase Assay System (Promega, Mannheim, Germany), Coomassie protein assay reagent (Pierce, Rockford, IL, USA), Trizol (Invitrogen, Carlsbad, CA, USA), chloroform, isopropanol, tetrahydrofuran, (Merck, Darmstadt, Germany), anti-eIF2α, anti-phospho-eIF2α (Cell Signalling, Boston, USA), anti-β-actin (Sigma, Taufkirchen, Germany), Proteostat PDI assay kit (Enzo, Lörrach, Germany), Cignal Lenti ERSE/ATF6/positive control Reporter (luc) kit (Qiagen, Düsseldorf, Germany). Chemiluminescence reagent was purchased from PerkinElmer LAS Inc. (Boston, MA, USA). Primers and all reagents were purchased for TaqMan PCR (ABI, Darmstadt, Germany). Secondary antibodies conjugated with horseradish peroxidase, anti-CHOP and anti-phospho-AMPK were purchased from Santa Cruz Biotechnology (Heidelberg, Germany).

### Cell culture

Human umbilical vein endothelial cells (HUVECs) were purchased from Promo Cell, Heidelberg, Germany and cultured in basal endothelial medium supplemented with 10% fetal bovine serum (FBS), essential growth factors and antibiotics. Cultures were maintained at 37°C, 5% CO_2_ humidified atmosphere and experiments were conducted on cells in passage 2–6 at approximately 80–90% confluence.

### Synthesis of N-acyl dopamines (NADD)

N-octanoyl dopamine (NOD), N-pivloyl dopamine (NPD) and N-octanoyl tyramine (NOT) were synthesized from commercially available precursors as previously described [20] and purified by twofold recrystallization from dichloromethane as demonstrated by thin layer chromatography (TLC). Octanoic or pivalic acid were converted to their mixed anhydride derivate by reaction with ethyl chloroformate in the presence of N-ethyl diisopropylamine. The crude mixed anhydrides were incubated with dopamine hydrochloride (Sigma-Aldrich, Taufkirchen, Germany) in N,N-dimethylformamide and N-ethyl diisopropylamine to form NOD or NPD. NOT was synthesized according to NOD using the analog mixed anhydride coupled to tyramine as reaction component. After aqueous preparation and evaporation of the organic solvent NOD in an overall yield of approximately 60% is obtained. The sample investigated by NMR (Bruker AC250) yielded spectra in accordance with the expected structure. Acetylation of NOD (A-NOD) was performed by suspending two grams of NOD in 5 ml acetic anhydride under magnetic stirring. When two drops of sulphuric acid were added, the suspension turned clear and stirring was continued for one hour. Diluted hydrochloric acid (5 mL) was added and 30 min later the reaction mixture was poured into 200 ml ice water. The precipitated product was collected by vacuum filtration and dried under vacuum to yield A-NOD, pure as judged by thin layer chromatography (TLC).

### Gene expression profiling

Sample preparation and processing was performed according to the Affymetrix GeneChip Expression Analysis Manual (http://www.Affymetrix.com). Total RNA was isolated from HUVECs using Trizol-Reagent (Life Technologies, Inc., Rockville, MD, USA). DNase treatment was carried out, using RNase free DNase I (Ambion, Woodward, Austin, TX, USA). RNA concentration and quality were assessed by RNA 6000 nano assays on a Bioanalyser 2100 system (Agilent, Waldbronn, Germany). 5 µg of RNA was converted into cDNA using T7-(dT)24 primers and the SuperScript Choice system for cDNA synthesis (Life Technologies, Inc., Rockville, MD, USA). Biotin-labelled cDNA was prepared by *in vitro* transcription using the BioArray high yield RNA transcript labeling kit (Enzo Diagnostics, Farmingdale, NY, USA). The resulting cDNA was purified, fragmented and hybridized to U133A gene chips (Affymetrix, Santa Clara, CA, USA). After hybridization the chips were stained with streptavidin–phycoerythrin (MoBiTec, Goettingen, Germany) and analysed on a GeneArray scanner (Hewlett Packard Corporation, Palo Alto, CA, USA).

### Microarray processing and statistical analysis

Gene expression profiling was performed using arrays of HG_U133A 2.0-type from Affymetrix. A Custom CDF Version 13 with Entrez based gene definitions was used to annotate the arrays. The Raw fluorescence intensity values were normalized applying quantile normalization. Differential gene expression was analysed OneWay-ANOVA using a commercial software package SAS JMP7 Genomics, version 4, from SAS (SAS Institute, Cary, NC). A false positive rate of α = 0.05 with FDR correction was taken as the level of significance. The raw and normalized data are deposited in the Gene Expression Omnibus database (http://www.ncbi.nlm.nih.gov/geo/; accession No. GSE- 56285).

### RNA isolation, PCR and RNA stability

Total RNA was isolated as described above. 1 µg of total RNA was reverse-transcribed into cDNA using the 1st Strand cDNA Synthesis Kit. cDNA was diluted in 20 µL DEPC-treated water and stored at −20°C until use. qPCR was performed on a ABI-Prism 7700 sequence detection system using TaqMan universal PCR master mix AmpErase UNG (part no. 4324018). The following Taqman assays were used: BiP (part No. Hs00607129_gH), CHOP (part No. Hs00358796_g1), PDIA4 (part No. Hs00202612_m1), ERO1L (part No. Hs00205880_m1) and GAPDH (part No. Hs02758991_g1). Samples were run under the following conditions: initial denaturation for 10 min at 95°C followed by 40 cycles of 15 s at 95°C and 1 min at 60°C. The levels of gene expression in each sample were determined with the comparative cycle threshold method. PCR efficiency was assessed from the slopes of the standard curves and was found to be between 90% and 100%. Linearity of the assay could be demonstrated by serial dilution of all standards and cDNA. All samples were normalized for an equal expression of GAPDH. For RT-PCR 1 µL of cDNA was amplified in a 20 µL reaction mix containing 2,5 mmol·L–1 dNTPs, 25 pmol·L–1 of each primer, 0,125 units Taq polymerase and 0,5 mmol·L–1 MgCl2. The following primers were used: : GAPDH forward: 5′- GTC TTC ACC ACC ATG GAG AA-3′ and reserve: 5′- ATC CAC AGT CTT CTG GGT GG-3′, Xbp1 forward: 5′- CCT TGT AGT TGA GAA CCA GG -3′ and reverse 5′-GGG GCT TGG TAT ATA TGT GG-3′. The cycling conditions used for various primers were as follows: 4 min of denaturation at 94°C, followed by 30 (Xbp1) or 25 (GAPDH) cycles of amplification, each consisting of denaturation for 30 s at 94°C, annealing for 30 s at 58°C (Xbp1) and 55°C (GAPDH) and extension for 1 min at 72°C. In all experiments GAPDH was used as housekeeping gene, no differences were found for the conditions tested when β-actin was used. After the last amplification a final extension for 10 min at 72°C was performed for each reaction. PCR products were analysed on a 4% NuSieve agarose gel containing ethidium bromide and run at 50 V for 6 hrs.

### Lentiviral transduction and reporter assays

To evaluate activation of UPR pathways, cells were transfected with commercially available reporter constructs as ready-to use transducer lentiviral particles (Qiagen, Düsseldorf, Germany). One day before transduction cells were seeded in 96-well plates (10^4^/well) and cultured overnight. Hereafter, the cells were transduced at an MOI of 5 using 8% polybrene. Five hrs after transduction 3 volumes of fresh cell growth medium was added and the cells were incubated overnight. Cell growth medium was replaced the next day and 24 hrs hereafter the cells were treated with NADD according to the specific experiment. Luciferase activity was evaluated by a commercially available luciferase assay system (Promega, Mannheim, Germnay) according to the manufacturer's protocol.

### Protein extraction and Western blot Analysis

HUVEC lysed in 20 mM Tris-HCl, 150 mM NaCl, 5 mM EDTA, 1% Triton X-100, 0.5% sodium deoxycholate, 1 µM dithiothreitol (DTT) buffer containing proteinase and phosphatase inhibitors. In some experiments, nuclear proteins were isolated as previously described [27]. Protein concentrations were measured using Coomassie-Reagent (Pierce, Rockford, USA). Samples (20 µg protein extract) were heated to 95°C for 5 minutes, loaded and separated on 10% SDS-polyacryamide gels followed by semi-dry blotting onto PVDF membranes (Roche, Mannheim, Germany). The membranes were incubated with 5% w/v non-fat dry milk or bovine serum albumin in TBS/Tween 0.5% to block unspecific background staining and hereafter incubated overnight at 4°C with the specific mono- or polyclonal eIf2α and p-eIf2α (Cell Signaling, Boston, USA), anti- CHOP and anti-pAMPK (Santa Cruz, Heidelebrg, Germany). Subsequently, the membranes were thoroughly washed with TBS-Tween 0.1% and incubated with the appropriate horseradish peroxidise conjugated secondary antibody (Santa Cruz, Heidelberg, Germany), followed by five times wash in TBS/Tween 0,1%. Proteins were visualized using enhanced chemo luminescence technology, according to the manufacturer's instructions (Pierce, Rockford, IL). To confirm equal protein loading, membranes were stripped and re-probed with monoclonal anti-β-actin antibody (Sigma, Taufkirchen, Germany).

### PDI activity assay

PDI activity was assessed in a test tube using a commercially available assay ProteoStat PDI assay kit (Enzo Life Sciences, Lörrach, Germany). The assay was performed as recommended by the manufacturer.

### FACS anaylsis

Cell apoptosis and cell cycle analysis were assessed by FACS. For cell apoptosis HUVEC were stimulated with NOD (100 µM) or Tunicamycin (1 µg/ml) for 24 hrs followed by Annexin V/PI staining (Life Technologies, Darmstadt, Germany). For cell cycle analysis HUVEC were stimulated with NOD (100 µM) for 24 hrs. After stimulation the cells were harvested by trypsin, washed three times with phosphate-buffered saline (PBS), and stained for 15 min at room temperature with 5 µl of Annexin V–FITC and 10 µl of PI (5 µg/ml) in 1 binding buffer (10 mm HEPES, pH 7.4, 140 mm NaOH, 2.5 mm CaCl_2_). For cell cycle analysis the cells were stained with DRAQ5 (Biostatus Lim., Shephsed, UK) at a final concentration of 10 µM according to the supplier's protocol. For all experiments cells were analyzed on a FACS Calibur flow cytometer (BD Biosciences, Heidelberg, Germany). At least 50,000 gated events were collected per sample and data were analyzed by Flowjo software (Tree Star, Inc., Ashland, OR, USA).

### Proliferation assay

HUVECs were seeded in multiple cell densities (2×10^3^–8×10^3^) in a 96-well plate and cultured in 100 µl of complete medium for 24 hrs. Hereafter cells were treated with different concentrations of NOD for 24 hrs and pulsed with 0.2 µCi of [6-3H] thymidine/well (Perkin Elmer, Groningen, The Netherlands) during the last 16 h of culture. All conditions were tested on six replicates culture wells. Incorporated 3Hthymidine was assessed by scintillation counting in a liquid scintillation counter (LS 6500, Beckman Coulter, Krefeld, Germany).

### Intracellular ATP measurement

Cells were cultured from passage 2 till passage 6 in the presence or absence of NOD (1 µM). At passage 6 the cells were seeded in 6-well plates and were cutured for additional 2days. Hereafter cells were harvested by trypsin, counted and 500.000 cells pro condition were lysed in 200 µl of lysis buffer (100 mM Tris, 4 mM EDTA, pH 7.7). Lysates were collected and ATP concentrations were assessed directly hereafter using a commercially available ATP-driven luciferase assay according to the manufacturer's instruction (Roche Diagnostics, Mannheim, Germany). All experimental conditions were tested in triplicates in at least 3 different experiments.

### LDH assay

Tolerance to hypothermia associated cell damage was assessed by lactate dehydrogenase (LDH) release. To this end, HUVEC were cultured from passage 2 till passage 6 in the presence or absence of NOD (1 µM). At passage 6 the cells were seeded in 24-well plates and were cultured for additional 2 or 5 days in the absence of NOD. Shortly before subjecting the cells to cold storage the medium was changed to phenol red free and cells were stored for 24 hrs at 4°C. Cell damage was assessed by LDH release in the supernatant, according to manufacturer's instructions (Roche diagnostics, Mannheim, Germany). All experimental conditions were tested in triplicate in at least 3 different experiments.

### Statistical analysis

All data are expressed as the means ± SD from at least three independent experiments. Statistical significance was assessed by one-way ANOVA (Dunnett's and Tukey test) and P<0.05 was considered to be significant.

## Results

### Induction of the UPR by NOD

To investigate if N-octanoyl dopamine (NOD) induces the UPR, we screened by genome wide gene expression profiling in HUVECs for genes that were up regulated by NOD. To this end, three different primary cultures of HUVECs were treated with 100 µM NOD for 24 hrs or left untreated. The results of the top 30 genes that showed up-regulation are depicted in Table 1. Thirteen out of these have been reported to be UPR target genes [3], [11], [28]. Up-regulation of the 5 most influenced UPR target genes was confirmed in independent experiments by qPCR (data not shown). The raw and normalized data are deposited in the Gene Expression Omnibus database (http://www.ncbi.nlm.nih.gov/geo/; accession No. GSE- 56285).

**Table 1:** Up-regulation of genes by NOD (NOD-treated vs. not treated cells).

Gene	Fold Change	p-value^a^
**FAM129A**	9,46	2.75
HMOX1	8,56	3.51
**DNAJB9**	8,22	3.97
**SLC3A2**	7,73	5.54
AKR1C1	7,37	2.25
**DDIT3**	6,69	3.89
IL13RA2	5,99	2.85
**ASNS**	5,68	2.39
**HERPUD1**	5,63	3.20
GDF15	5,26	3.21
SQSTM1	4,58	3.16
RRAGD	4,52	2.31
**HYOU1**	4,36	2.52
IL8	4,20	4.22
**PDIA4**	4,17	2.19
LBH	3,94	2.57
BLVRB	3,92	2.52
WARS	3,79	2.27
**BiP**	3,54	2.41
SDF2L1	3,46	3.17
**TRIB3**	3,44	2.64
P4HA2	3,38	3.39
**MANF**	3,27	2.83
**SEL1L**	3,24	2.31
CTNS	3,23	3.12
CCRL2	3,15	4.10
TM6SF1	3,08	2.48
**DERL2**	3,08	2.70

a : **p**-values for the comparison are given as log10 value. UPR target genes are depicted in bold.

**Figure 1:**
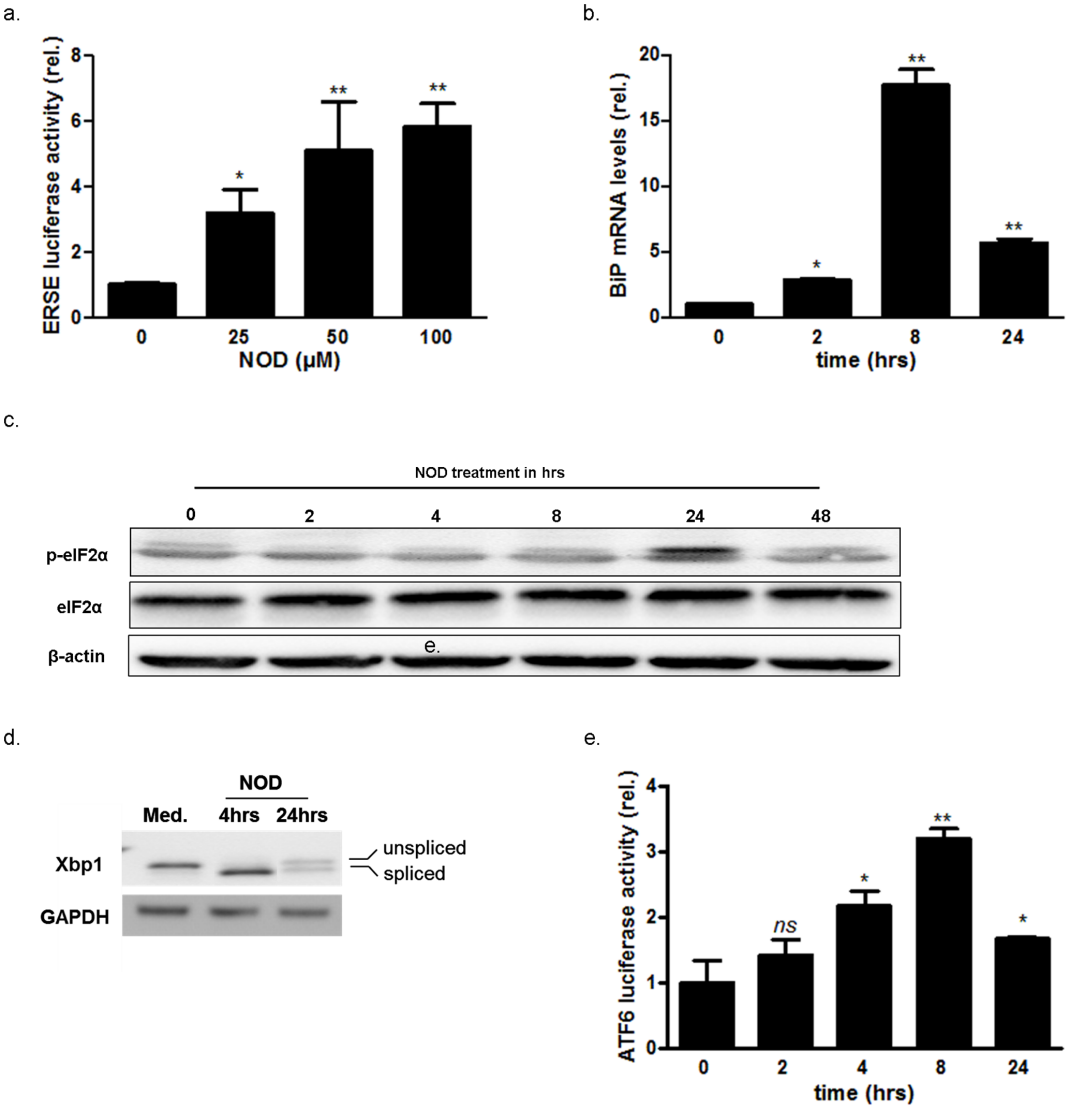
Induction of the UPR by NOD. (a) HUVECs were transduced by lentiviral particles containing either an ERSE- luciferase reporter or a luciferase construct under control of a CMV driven promoter as transduction efficiency control. The cells were stimulated for 8 hrs with 25 µM, 50 µM and 100 µM of NOD as indicated. Luciferase activity was assessed as described in materials and methods and normalized for constitutively expressed luciferase. The results are expressed as normalized ESRE luciferase activity relative to untreated (0 µM) cells. Values represent mean ± SD from three independent experiments. *P<0.05, **P<0.01 vs. untreated control. (b) HUVECs were treated with 100 µM NOD for the indicated time periods. Total RNA was isolated and the expression of BiP and GAPDH was assessed by qPCR. The results are expressed as BiP mRNA levels, normalized for GAPDH and relative to cells that were not treated. *P<0.05, **P<0.01 vs. untreated control. (c) HUVECs were treated with 100 µM NOD for the indicated time periods. Hereafter protein extracts were made and phosphorylation of eIF2α was assessed by Western blotting. The blots were stripped and re-probed with antibodies directed against total eIF2α and β-actin to test for equal loading. The results of a representative blot are depicted, a total of 4 independent experiments were performed. (d) HUVECs were treated for 4 and 24 hrs with 100 µM of NOD before total RNA was isolated. Cells that were not treated (Med.) served as control. Spliced and unspliced xbp-1 mRNA were detected by PCR. The spliced and unspliced amplification products differ by 26 bp. (e) Cells were transduced with lentiviral particle containing an ATF-6-driven luciferase reporter or a luciferase construct under control of a CMV driven promoter as transduction efficiency control. The cells were stimulated for different time periods with NOD 100 µM. Luciferase activity was assesses as described in materials and methods and normalized for constitutively expressed luciferase. The results are expressed as normalized ATF6 luciferase activity relative to timepoint 0. Values represent mean ± SD from three independent experiments. **P<0.01, * P<0.05 vs. untreated control, *ns:* no significant.

To further substantiate our affymetrix findings and to confirm that NOD treatment induces the UPR we made use of a cis acting ER-stress response element (ERSE) - containing luciferase reporter construct. Luciferase expression increased upon NOD treatment in lentiviral transduced HUVEC in a dose-dependent fashion (Figure 1a). Since these cis acting elements are present in the promoter of BiP, we assessed the influence of NOD on BiP mRNA expression. In line with the ERSE reporter assay it was found that BiP mRNA transiently increased upon NOD treatment, with maximal expression occurring at 8 hrs of stimulation (Figure 1b).

NOD did not selectively activate one particular proximal UPR sensor as PERK, IRE1 and ATF6 were all transiently activated. Phosphorylation of eIF2α was in most experiments evident after 24 hrs of NOD treatment. In some experiments phosphorylation was also noted at 8 hrs, but was persistently absent at later time points (Figure 1c). Similarly, IRE1 and ATF6 were transiently activated upon NOD treatment. Xbp1 splicing (Figure 1d) and ATF6 induced luciferase expression (Figure 1e) were maximal at 4 and 8 hrs respectively, with decreasing tendency at later time points.

### Structural requirements of NOD for UPR induction

**Figure 2:**
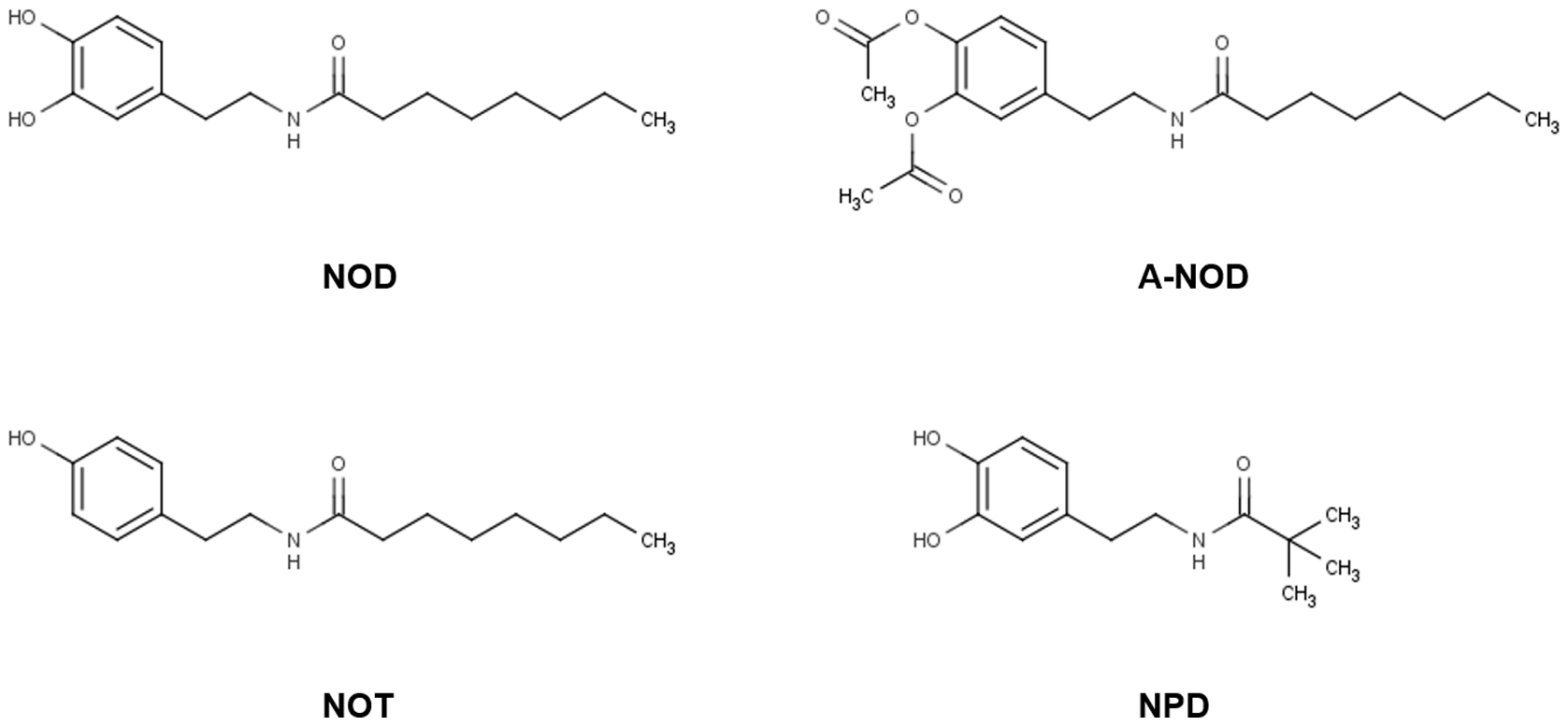
Molecular structure of NADD used in the study.

**Figure 3:**
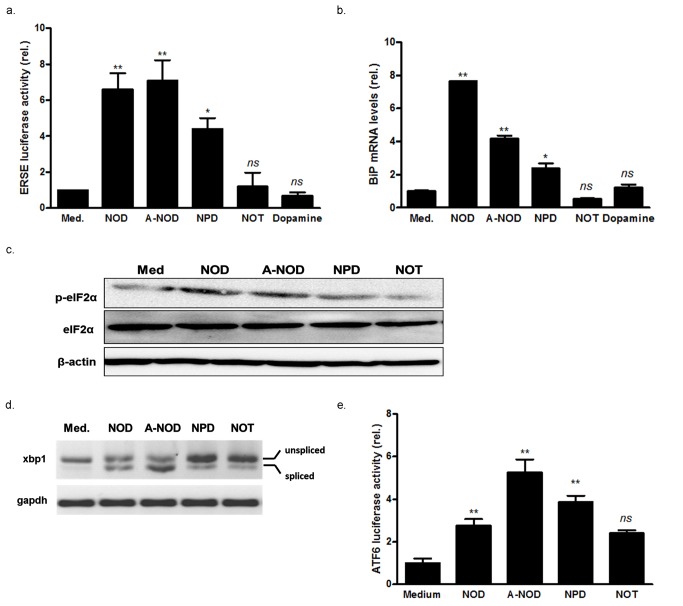
Induction of the UPR by NADD variants. (a) HUVECs were transduced by lentiviral particles containing either an ERSE- luciferase reporter or a luciferase construct under control of a CMV driven promoter as transduction efficiency control. The cells were stimulated for 8 hrs with 100 µM of different NADD as indicated. Equimolar concentrations of dopamine were also included to assess if dopamine induces the UPR. ERSE dependent luciferase activity was assessed as described in materials and methods and normalized for constitutively expressed luciferase. The results are expressed as normalized ESRE luciferase activity relative to untreated (Med.) cells. Values represent mean ± SD from three independent experiments, *P<0.05, **P<0.01 vs. untreated control, *ns:* not significant. (b) HUVECs were treated with 100 µM NADD for 8 hrs. Cells that were not treated (Med.) served as control. Hereafter total RNA was isolated and the expression of BiP and GAPDH were quantitated by qPCR. The results are expressed as BiP mRNA levels, normalized for GAPDH mRNA expression and relative to cells that were not treated (Med), *P<0.05, **P<0.01 vs. untreated control, *ns*: not significant. (c) HUVECs were stimulated for 8 hrs with 100 µM of different NADD, or left untreated (Med). Hereafter protein extracts were made and phosphorylation of eIF2α was assessed by Western blotting. The blots were stripped and re-probed with antibodies directed against total eIF2α and β-actin to test for equal loading. The results of a representative blot are depicted, a total of 4 independent experiments were performed. (d) HUVECs were stimulated for 8 hrs with different NADD (100 µM) before total RNA was isolated. Cells that were not treated (Med.) served as control. Spliced and unspliced xbp-1 mRNA were detected by PCR. The spliced and unspliced amplification products differ by 26 bp. (e) Cells were transduced with lentiviral particle containing an ATF-6-driven luciferase reporter or a constitutively expressed luciferase construct. The cells were stimulated for 8 hrs with different NADD (100 µM). Luciferase activity was assessed as described in materials and methods and normalized for constitutively expressed luciferase activity. The results are expressed as normalized ATF6 luciferase activity relative to untreated (Med.) cells. Values represent mean ± SD from three independent experiments, *P<0.05, **P<0.01, vs. untreated control, *ns*: no significant.

To assess the structural requirements for UPR induction by NOD we used NOD as lead compound and subsequently changed the functional groups at the aromatic ring or exchanged the octanoyl - for the more bulky pivaloyl moiety. The changes at the aromatic ring included, omission of one hydroxyl group (N-octanoyl tyramine (NOT) and acetylation of the hydroxyl-groups (A-NOD). While NOT has a significant lower redox activity compared to NOD, in A-NOD oxidation of the masked catechol structure can only occur intracellular. Moreover acetylation of the hydroxy groups makes the molecule more polar which may facilitate cellular uptake. The presence of the bulky pivaloyl moiety in N-pivaloyl dopamine (NPD) makes the ester more resistant to hydrolysis [29] (Figure 2). Using the ERSE- containing luciferase reporter construct, it was shown that only the redox active compounds NOD, A-NOD and to a lesser extent NPD were able to increase luciferase expression in lentiviral transduced HUVEC, while the redox inactive NOT showed no effect (Figure 3a). In line with these findings, increased BiP mRNA expression and ER-sensor activation (Figure 3b–e) only occurred by the redox active NADD variants. It should be mentioned however that with exception of the ATF6 reporter assay, in all other assays NPD was consistently weaker compared to NOD and A-NOD. In contrast to the NADD variants, equimolar concentrations of dopamine were not able to activate the UPR (Figure 3a and b).

**Figure 4:**
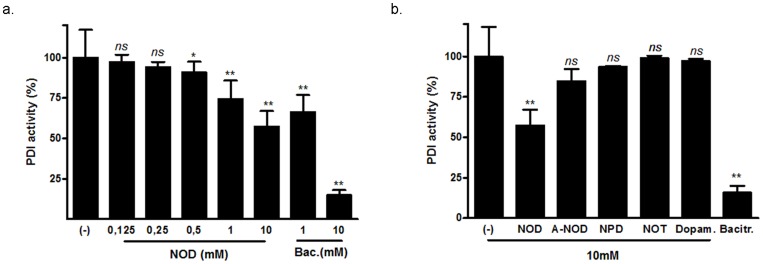
NOD inhibits PDI activity. (a) PDI activity was assessed by monitoring PDI-catalyzed reduction of insulin in the presence of Dithiothreitol (DTT) and different concentrations of NOD. Bacitracin in concentrations of 1 and 10 mM was used to validate the assay. (b). Different NADD were tested for inhibition of PDI activity in a similar manner as described in (a). The results from three independent experiments are expressed as mean % PDI activity ± SD, using PDI activity in the absence of inhibitors (−) as 100%, *P<0.05, **P<0.01 vs. untreated control, *ns*: no significant.

Since oxidative folding in the ER is catalyzed by protein disulfide isomerase (PDI) we assessed in a test tube assay if PDI activity was impaired by NOD. The known PDI inhibitor bacitracin was included to validate the assay and showed inhibition of 65 and 15% at a concentration of 1 and 10 mM respectively (Figure 4a). Although NOD was clearly less effective compared to bacitracin, NOD inhibited PDI in a dose dependent manner with an almost 50% inhibition at 10 mM. A-NOD and NPD did not inhibit PDI activity (Figure 4b).

### NOD does not impair cell viability

**Figure 5:**
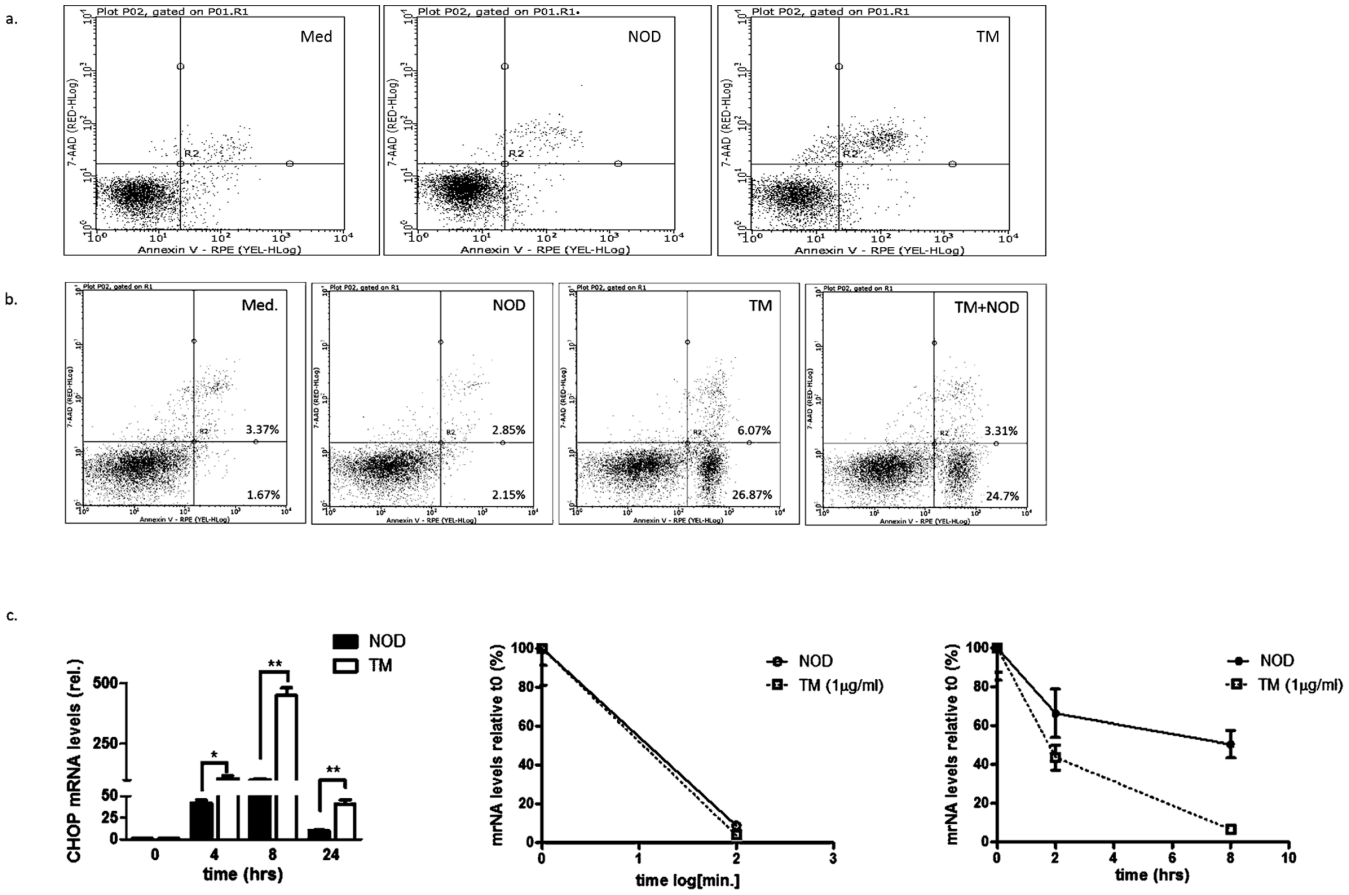
NOD does not impair cell viability. (a) HUVECs were treated for 24 hrs with NOD (100 µM) or tunicamycin (TM, 1 µg/ml). In addition cells were treated for 3 hrs with 100 µM of NOD prior to tunicamycin treatment. Cells that were not treated (Med.) served as control. Both floating and adherent cells were harvested, stained for Annexin V and 7-AAD and analysed by flow cytometry. (b) HUVECs were treated with 100 µM NOD or TM (1 µg/ml) for the indicated time periods. Total RNA was isolated and expression of CHOP mRNA was assessed by qPCR and normalized for GAPDH (left panel). Cells were pre-treated either with NOD or with tunicamycin and then chased for 0, 2, 4 and 8 hrs with AcD 50 ng/ml. Total RNA was isolated and the expression of CHOP (middle panel) and BiP (right panel) mRNA were assessed by qPCR and normalized for GAPDH. The results of three different experiments are expressed as mean mRNA levels ± SD, ** *P*<0.01 vs. untreated cells.

In keeping with the fact that induction of the UPR may result in apoptosis, we sought to address if NOD treatment affects cell viability. Cell viability was assessed by means of FACS analysis using Annexin V/7-ADD (Figure 5a). While tunicamycin, a known UPR inducer, clearly increased the percentage of 7-AAD single and Annexin V/7-ADD double positive cells, this was not observed for NOD (Figure 5a). Interestingly, when HUVEC were treated with NOD 3 hrs prior to the addition of tunicamycin, the number of both 7-AAD single and Annexin V/7-AAD double positive cells decreased (Figure 5a). Transient induction of CHOP mRNA was observed for both NOD and tunicamycin, albeit that CHOP mRNA expression was significantly higher for the latter condition (Figure 5b, left panel). No significant difference in CHOP –mRNA stability was observed between NOD and tunicamycin treated cells (Figure 5c, middle panel). However, BiP mRNA was more stable in NOD treated cells than tunicamycin-treated cells (Figure 5c, right panel).

### Growth arrest, hypometabolism and thermotolerance

**Figure 6:**
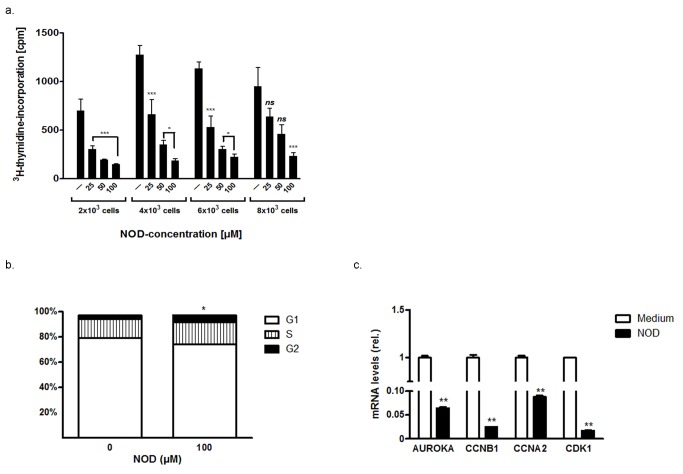
NOD inhibits cell proliferation. (a) HUVECs were seeded in different cell densities in 96 well plates and the next day treated for 48 hrs with different concentrations of NOD. During the last 16 hrs the cells were pulsed with 0.2 µCi 3[H] thymidine and subsequently harvested. The results of a representative experiment are depicted and expressed as mean 3H-thymidine incorporation in counts per minute (cpm) ± SD of 6 replicate wells for each condition. A total of 6 experiments were performed with essentially similar results. Significance was defined as *P<0.05 and **P<0.01 compared to untreated cells, *ns*: not significant. (b) In separate experiments HUVECs were stimulated with 100 µM of NOD or left untreated. One part of the cells was used for cell cycle analysis, for the other part total RNA was isolated and the expression of AUROKA, CCNB1, CCNA2 and CDK1 was assessed by qPCR (c). A total of three experiments were performed. The results in graph (b) are expressed as mean percentage of cells in G0/1-, S- and G2/M- phase. *P<0.05, NOD vs. untreated. The results in graph (c) are expressed as normalized mRNA levels relative to the untreated medium control cells. ** *P*<0.01 vs. untreated control.

In the course of performing this study we consistently observed that the time to confluence was much longer when HUVEC were seeded in culture plates and treated with NOD. Consequently we assessed if NOD affects cell proliferation. As demonstrated in ^3^[H] thymidine incorporation assays, NOD dose dependently inhibited cell proliferation (Figure 6a). NOD treatment changed cell cycle phase distribution, with a relative increase of cells in the S-G2/M phase (Figure 6b), suggesting that cell cycle progression was attenuated at the S-G2/M phase. Importantly, affymetrix analysis also showed a significant decreased mRNA expression for genes involved in S-G2/M progression (table 2), which was confirmed by qPCR (Figure 6c).

**Table 2:** Genes implicated in cell cycle, down-regulated by NOD.

Gene	Fold Change	p-value^a^
CDC20	9.35	2.32
CCNB1	8.48	2.77
CDK1	8.47	2.84
PRC1	8.08	2.30
NUSAP1	7.67	2.75
AURKB	7.26	3.65
NDC80	6.40	3.64
KIF4A	6.27	2.59
CEP55	6.22	2.38
CCNA2	6.15	3.88
CENPE	5.45	2.60
CDKN3	5.31	2.75
SPC25	5.09	2.91
AURKA	4.84	3.14
CDCA8	4.82	9.80

a : **p**-values for the comparison are given as log10 value.

**Figure 7:**
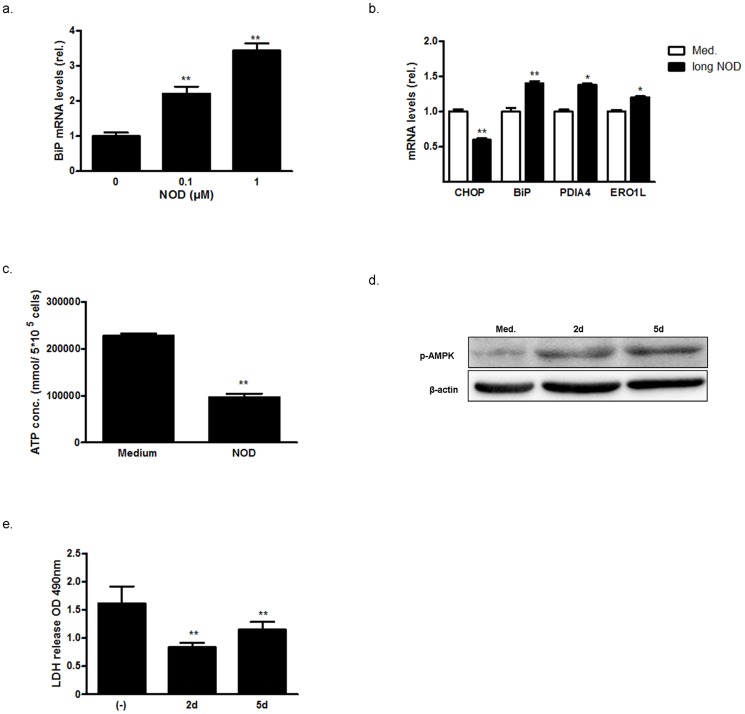
Long-term NOD treatment results in hypometabolism and thermotolerance. Three different primary HUVEC isolations were cultured from P2 until P6 in the absence or presence of the indicated NOD concentrations. (a) BiP mRNA expression for cells cultured in the presence of 0.1 and 1 µM NOD was assessed by qPCR. (b) CHOP, BiP, PDI4, and ERO1L mRNA expression for cells cultured in the presence of 0.1 µM NOD was assessed by qPCR. The results in a and b are expressed as normalized mean mRNA levels relative to untreated controls (0, Med) ± SD of 3 replicates for each condition and for all 3 HUVEC isolations. ** *P*<0.01, *P<0.05 vs. untreated cells. (c) Intracellular ATP concentration was assessed in HUVEC at P6 upon long-term culture in the absence (medium) or presence of NOD (1 µM). The results are expressed as mean ATP concentration (pmol/5*105 cells) ± SD of 3 replicates for each condition and for all 3 HUVEC isolations. **P<0.01 vs. untreated cells (d,e). In separate experiments NOD (1 µM) containing medium was exchanged for normal cell culture medium at P6 and the cells were cultured for 2 (2d) or 5 (5d) days in the absence of NOD. Cells that were continuously cultured in normal cell culture medium (Med.) were used as control. In (d) protein extracts were made and assessed for phosphorylation of AMPK (p-AMPK) by Western blotting. The blots were stripped and re-probed with antibodies directed against β-actin to test for equal loading. The results of a representative blot are depicted, a total of 3 independent experiments were performed. In (e) the cells were subjected to cold storage (4°C) for 24 hrs. Hereafter LDH release was assessed in the supernatants. Each condition was tested in triplicate in 2 independent experiments. The results are expressed as mean LDH release (OD490) ± SD, **P<0.01 vs. (Med.) control.

With exception of CHOP, all other UPR target genes tested in this study were up-regulated in a dose dependent manner when HUVEC were treated with low concentrations of NOD (0.1 and 1 µM) form the 2^nd^ passage on until the 6^th^ passage (P2 to P6) (Figure 7a and b). In cells that were treated over this long period of time with NOD, intracellular ATP concentrations were significantly lower as compared to cells from a similar passage without treatment (Figure 7c). The lower intracellular ATP concentration was paralleled by an increased phosphorylation of AMPK (Figure 7d), and was still noted when NOD was removed from the culture 2 or 5 days prior to analysis. In addition to this apparent hypo-metabolic state, cells were also more resistant to cold inflicted injury as determined by LDH release (Figure 7e)

## Discussion

In the present study we demonstrate that synthetic NADD transiently activate the UPR. This property seems to be dependent on the redox activity of these compounds. NOD did not affect cell viability, but strongly impaired cell proliferation of HUVEC, most likely by attenuation of cells in the S-G2/M phase. In concordance to this, mRNA expression for a number of genes involved in S-G2/M progression was significantly down-regulated by NOD. Interestingly, long-term NOD treatment resulted in hypometabolism and thermotolerance, as suggested by a decreased intracellular ATP concentration, activation of AMPK and increased resistance to cold inflicted cell injury.

Sensing, responding, and adapting to environmental insults are essential for all living organisms and are originating from evolutionarily conserved unique signal transduction pathways. Being one such a pathway, the role of the UPR is to alleviate ER stress and, paradoxically, to activate apoptosis, depending on the nature and severity of the stressor [30]. NADD are able to activate the UPR mainly due to their redox active catechol structure, albeit that at high NOD concentrations (10 mM) PDI activity was also inhibited in a test tube assay. The latter seems to be a highly unlikely explanation for UPR induction in HUVEC, since low NOD concentrations (25 µM) already induced the UPR. It is therefore more conceivable that oxidation of the catechol containing NADD leads to donation of reduction equivalents, thereby changing an oxidizing ER environment to a more reducing one. This assumption is supported by the finding that for NOT, which contains a low redox active tyramine moiety, UPR induction was not observed. It should also be mentioned that the hydrophobic fatty acid of NADD may facilitate UPR induction by providing an easier access to the ER, in line with the observation that despite its catechol structure dopamine was not able to induce the UPR. We are aware that no definite proof that NOD indeed has access to the ER is provided. However previous published studies in which dopamine uptake was compared to that of NOD [20] revealed that both dopamine and NOD were found in the membrane fraction, which predominantly contains the ER compartment, as well as in the mitochondrial- and cytosolic fraction. Hence, NOD may also impair the redox milieu in other cellular compartments than the ER. It should be emphasized that the ER enriched fraction also contained small amount of mitochondrial proteins, and hence formal proof that NOD has access to the ER compartment is still lacking.

The lower efficacy of NPD for UPR induction might reflect a more limited access of NPD to the ER as a consequence of the bulky pivanoyl fatty acid. Recently, Achard et al demonstrated that the long-chain saturated palmitate (C16:0) fatty acid activates the UPR [31], questioning as to whether UPR induction as observed in our study could be mediated via intracellular hydrolysis of NADD. This scenario is however unlikely because hydrolysis of NOT would result in the release of octanoic acid (C8:0), yet UPR induction was not observed when equimolar concentrations of NOT were applied to HUVEC. The concentrations of palmitate used by Achard et al were significantly higher compared to concentration of NOD at which UPR induction was already noted (750 vs. 25 µM).

The decision mechanisms for switching the UPR from adaptive response to apoptosis have so far been poorly understood. Cojocari et al [32] recently suggested that the PERK signaling arm is uniquely important for promoting adaptation and survival during hypoxia-induced ER stress. Yet, other reports suggest that UPR mediated cell adaptation is not due to selective UPR sensor activation, but most likely reflects changes in protein composition or mRNA stability of adapted cells [33]. Compared to tunicamycin, NOD mediated induction of both CHOP and BiP mRNA was modest, and may explain why tunicamicin, but not NOD treated HUVEC went into apoptosis. Interestingly, in long-term NOD (1 µM) treated HUVEC CHOP mRNA was down-regulated while that of BiP was persistently up-regulated. This is in line with the observation by Rutkowski et al [33] that over a long time course of low ER stress CHOP up-regulation diminishes, despite persistent up-regulation of the UPR-responsive ER chaperones BiP. It remains to be assessed why prior NOD treatment of HUVEC, abrogates tunicamycin mediated apoptosis, but it may well be a consequence of altered UPR signaling [33]–[36].

Similar as reported by Rutkowski et al for tunicamycin [33], we also observed that cell growth was retarded when cells were continuously cultured in the presence of low NOD concentrations, as suggested by an increased time to confluence. It has been reported that phenolic compounds significantly affect cell proliferation by arresting cells in the G2/M phase [37]–[40]. Therefore it is at present unclear if growth inhibition by NOD is causally related to UPR induction or if this is independent of the UPR.

AMP-activated protein kinase (AMPK) is a cellular energy sensor that responds to low endogenous energy by stimulating fatty acid oxidation. It acts as a low-fuel warning system that is activated by depletion of ATP or, alternatively, by increased levels of AMP, to induce an energy-saving state and to prevent lactate accumulation and cell injury [41]. Since long term NOD treatment resulted in decreased intracellular ATP concentrations it is conceivable that this might have triggered the activation of AMPK as observed in our study. More importantly, we also observed that long term NOD treated HUVEC were more resistant to cold inflicted injury. This effect was still apparent when NOD was removed from the culture medium and cells were grown for additional 2 or 5 days. These findings have important implications as pre-conditioning strategies to improve organ functioning after cold storage are primarily focused on influencing cellular metabolism or energy status and reduction of inflammation [42]. Mamady H et al, have suggested that the UPR might be integral to long term survival in a state of cold torpor by coordinating gene expression responses that support the hibernating phenotype [14]. However, it would be prudent to be cautious in concluding that the apparent hypometabolism and thermotolarance mediated by NOD are linked to UPR induction.

We have previously demonstrated that NOD has therapeutic efficacy in ischemia induced acute kidney injury [19]. Induction of the UPR has been suggested to be protective in a variety of ischemia/reperfusion models [43]–[45]. It therefore remains to be assessed if therapeutic concentrations of NOD are able to activate UPR in vivo and to what extent this contributes to the reno-protective effect of NOD in ischemia induced AKI.

NADD were first described as potent inhibitors of 5-lipoxygenase [46], [47] and through the discovery that NADD are highly expressed in brain tissue, subsequent function have been revealed. Now these conjugates have come to prominence because of their potential roles in the nervous system, vasculature and the immune system and are being explored as potential lead compounds in drug development. The present finding that NADD have the propensity to induce hypometabolism and thremotolerance, irrespective as to whether this is mediated by UPR induction, opens new perspectives in this regard.
